# Pharmacological Treatment for the Management of COVID-19: A Narrative Review

**DOI:** 10.31729/jnma.5920

**Published:** 2021-06-30

**Authors:** Neelam Dhakal, Anil Poudyal, Pradip Gyanwali

**Affiliations:** 1 Nepal Health Research Council, Ramshah Path, Kathmandu, Nepal

**Keywords:** *COVID-19*, *pharmacological treatment*, *vaccines*

## Abstract

Severe acute respiratory syndrome coronavirus 2 infections are alarming worldwide incurring tremendous loss of life and possession. Individuals are facing a terrible pandemic condition in the absence of appropriate medicines to combat severe acute respiratory syndrome coronavirus 2 infections. This review aimed to provide details on pharmacological treatment measures that were adopted to fight COVID 19 pandemic globally including Nepal. A massive review was performed including 110 articles from the relevant field. Preliminary results on the efficacy of some existing antiviral agents were found, however, promising data on effective treatment regimen for COVID 19 are yet to be obtained. This review examines various drugs, their mechanism of actions and vaccines which are currently being used in clinical trials or are recommended for use in COVID 19 infection.

## INTRODUCTION

SARS-CoV-2 or COVID-19 is a novel virus belonging to the family coronavirus. It started from Wuhan, China on December, 2019 and was recognized as a pandemic on 11^th^ March, 2020 by the World Health Organization (WHO).^1^ Globally, 16,50,96,424 people are infected due to SARS-CoV-2 causing more than 34,22,417 deaths as of 19^th^ May , 2021.^2^ Despite of extensive efforts all around, a complete SARS-CoV-2 treatment protocol is yet to be identified leaving the pandemic an unexplained mystery. The pandemic began in Nepal from 23^rd^ January after the first case of SARS-CoV-2 was detected^3^ and 4,80,418 individuals have been infected, causing 5,657 deaths by the 19^th^ May, 2021. Currently, Nepal is witnessing a second wave of pandemic resulting in more than 8000 new cases and around 200 deaths per day.^4^ This analysis was therefore intended to provide information on possible pharmacological therapy that is being researched or used internationally to treat COVID-19.

## LITERATURE SEARCH

The narrative review was carried out from December 2020 to May 2021. In total, 150 relevant papers were downloaded and analyzed. After comprehensive study, 110 articles were selected for analysis.

Articles published between January and October 2020 was included. Relevant documents, guidelines, and information were obtained from the website of Ministry of Health and Population, Department of Drug Administration, Nepal Medical Council, Nepal Health Research Council and World Health Organization. Google Scholar and Pubmed databases were used to search the literature. For the literature quest, Boolean operators "And" and "OR" were used to ensure that all the applicable literature was not overlooked. The term "SARS-CoV-2", "COVID 19" and "corona virus" were used. The keywords "Convalescent plasma", "Remdesivir", "Favipiravir", "pharmacological treatment" and "arbidol" were combined with "SARS-CoV-2", "COVID 19", "corona virus", "COVID 19 vaccines" and "Corona vaccines". All the selected literatures were coded and information collected from each article was organized in Microsoft Office Excel 2007. The obtained information was categorized into several subjects and summarized.

## FINDINGS MECHANISM OF ACTION OF SARS-COV-2

Unlike other coronaviruses, SARS-CoV-2 infects respiratory tract and gastrointestinal tract of human beings and their major targets for invasion include epithelial cells of nasal, alveoli of the lungs, enterocytes of the bowel and pneumocytes.^5^ Corona virus has been classified into four genera which includes: alpha-CoV, beta-CoV, gamma-CoV, and delta-CoV(2). SARS-CoV and SARS-CoV-2 recognize Angiotensin Converting enzyme-2 receptors in human beings.^6,7,8^ CoV binds to the receptor through its spike glycoprotein (S) which has two subunits S1 and S2. S1 mediates attachment to the receptor while S2 is responsible for fusion of the membrane.^9^ Phylogenetic analysis of the virus showed that the novel virus is lineage B beta CoV.^10^ CoV S fall under Class I viral fusion protein category which requires protease cleavage for activation of its potential for fusion. One or more than one host proteases might cleave CoV S which depends on the strains of virus and cell types. Based on the protease available in the target host cells, CoV S might enter the cell either through plasma membrane or endocytosis.^11-17^

## POTENTIAL DRUGS AGAINST SARS-COV-2 ANTIVIRAL AGENTS

A study carried out molecular docking of Ribavirin, Remdesivir, Sofosbuvir, Tenofovir, Galidesivir against SARS-CoV-2 and showed higher binding affinity of these drugs to RNA dependent RNA polymerase (RdRp) of COVID-19. These antiviral agents are anti-RdRp drugs which bind tightly to RdRp site of COVID-19 and thus suggested for the treatment and management of COVID-19.^18^ Other antiviral agents like Ritonavir, Lopinavir, Remdesivir were also studied against SARS-CoV-2 nonetheless, in vitro analysis and animal study model revealed Remdesivir as better potential antiviral agents than Lopinavir and Ritonavir to treat SARS-CoV-2.^19-22^ Based on the pre-clinical data that support anti-viral activity against coronaviruses (SARS and MERS) and the in vitro ability of the agents to inhibit SARS-CoV-2 replication, medicines like were hydroxychloroquine, azithromycin, ritonavir, ruxolitinib, and camostat suggested and proceeded for clinical trials.^5^ Other nucleoside/nucleotide analogues like sofosbuviralovudine and zidovudine, have also been found to be active against the SARS RdRp in in-vitro biochemical assays and could have the potential to be repurposed against COVID19.^23^ Recently, there is increased attention towards use of Remdesivir and Favipiravir against SARS-CoV-2 and clinical trials are being carried out in different countries including Nepal. Studies have shown significant benefits with the use of Remdesivir and Favipiravir in the treatment of SARS-CoV-2.^24,25^

## REMDESIVIR

Remdesivir, which was developed against Ebola virus, possesses a broad spectrum antiviral property against many RNA viruses. Remdesivir is a prodrug of nucleotide analogue which exhibits inhibition property of viral RNA dependent RNA polymerase (RdRp)^18^ and is found to have both prophylactic and therapeutic efficacy against SARS-CoV-2. Remdesivir was found to improve pulmonary function, decrease viral load in lungs and also reduce several lung pathologies and show superior antiviral activity compared to Ritonavir and Lopinavir. In a trial conducted at 105 hospitals in the United States, Europe, and Asia provided little evidence regarding potential efficacy of Remdesivir in patients with moderate COVID-19 in 5 day course.^26^ Few studies showed improvement in oxygen-support status with the use of Remdesivir among severe COVID 19 patients.^27,28^ Surprisingly, a randomised, double-blind, placebo-controlled, multicentre trial carried out among adults with severe COVID-19 at ten hospitals of Hubei, China found no clinical benefits of Remdesivir.^29^ On World Health Organization (WHO) published a notice mentioning that there is no use of Remdesivir in COVID 19 infection.^30^

The Food and Drug Administration (FDA) has approved Remdesivir for the treatment of COVID-19 in hospitalized adult and pediatric patients over the age of 12 and weighing less than 40 kg. It is also approved by the FDA for the treatment of COVID-19 in hospitalized pediatric patients weighing 3.5 kg to 40 kg or aged below 12 years and weight more than 3.5 kg.^31^ Likewise, an observational study carried out by Nepal Health Research Council had showed significant improvement in COVID patients with the use of Remdesivir. The study showed 98.4% survival rate with the use of Remdesivir among moderate COVID patients. Similarly, better outcome was seen in serious COVID patients with the use of Remdesivir in Nepal along with minimal adverse effects (4.5%).^32^

## MECHANISM OF ACTION OF REMDESIVIR

Remdesivir is a prodrug that gets metabolized into an alanine metabolite as it enters into the cell and then later ultimately gets converted into active nucleoside triphosphate derivative (NTP). Nucleoside triphosphate can be utilized mistakenly by viral RNA dependent RNA polymerase for replication and NTP then gets incorporated within viral RdRp and interferes primarily by delaying the chain termination. Inhibitory action in viral replication was also associated with Remdesivir.^20,22,33,34^

## FAVIPIRAVIR

Favipiravir is an antiviral agent and is considered as a drug of choice against influenza virus. It selectively inhibits RNA dependent RNA polymerase of influenza virus. Evidences show effective outcome of Favipiravir in the treatment against influenza and Ebola virus.^25,35-37^ In an open label comparative controlled study carried out among COVID-19 patients demonstrated that patients treated with Favipiravir were found to have faster clearance of virus and changing of chest imaging compared to the patients treated with Ritonavir and Lopiravir.^38^A study conducted in China showed that efficacy of Favipiravir in severe COVID-19 patients was less than arbidol in day 7 while, it was found effective in moderate COVID-19 patients than arbidol.^39^ A clinical trial of Favipiravir among patients with moderate COVID 19 infection is going on in Nepal at present.

## MECHANISM OF ACTION OF FAVIPIRAVIR

Favipiravir is a synthetic prodrug which when reached to the tissue gets phosphoribosylation to active favipiravir-RTP. This active molecule acts as a substrate of RNA dependent RNA polymerase enzyme of virus and gets bind to it thus terminating protein synthesis process in virus.^40^

## ARBIDOL

Arbidol is an antiviral agent that possesses broad spectrum activity which has been effective against influenza and many other viral infections. Arbidol is a licensed drug for the treatment of influenza and many viral infections in Russia and China.^41^

In a study carried out to analyze efficacy and safety of lopinavir and ritonavir and arbidol monotherapy among 36 and 16 COVID-19 patients respectively, no viral load was found after 14 days of administration of arbidol while, viral load was detected in 15 COVID-19 patients after 14 days of administration of lopinavir and ritonavir.^42^ Likewise, another study carried out in china among severe COVID-19 patients found that Arbidol was more effective for clinical rate recovery of COVID-19 patients than Favipiravir in day 7. Favipiravir was found to be associated with decrease pyrexia and cough.^39^

However, another study has revealed only little benefit of using arbidol or Ritonavir/ Lopinavir among COVID-19 patients with mild to moderate symptoms.^28^

## MECHANISM OF ACTION OF ARBIDOL

Primary mechanism of action of arbidol is through blocking of viral fusion with the cell membrane of host which prevents entry of the viruses into the cell.^43^ A study through molecular docking showed that arbidol can be effective against COVID-19 possibly by blocking the trimerization of SARS-CoV-2 spike glycoprotein. This blocking makes the virus less infectious.^44^

## OTHER MEDICINES CHLOROQUINE AND HYDROXYCHLOROQUINE

Chloroquine is commonly used as antimalarial and anti-rheumatic drugs and was found effective against Ebola virus, chikungunya virus, dengue virus in laboratories studies but no effective result was obtained in clinical studies.^45-48^ Similarly, chloroquine was also reported to inhibit replication of SARS-CoV, MERS-CoV and SARS-CoV-2 in several studies^49-54^ except two studies which showed no significant inhibitory effects of chloroquine on MERS-CoV and hepatitis virus (MHV4).^55,56^ Thus, chloroquine and hydroxychloroquine were proposed for the treatment of COVID-19 at early phase of pandemic. However, clinical trials showed no significant effect on use of chloroquine instead adverse effects like arthralgia was observed.^47,57-59^

## CORTICOSTEROIDS

Corticosteroids exhibit anti-inflammatory effects and are drugs of choice in respiratory infections. Thus, they were also considered as a potential therapeutic agent in COVID-19 at early days of pandemic but, contradictory results were obtained with the use of corticosteroids in COVID-19. Delay in clearance of viral infections was observed with the use of corticosteroids among COVID-19 patients along with the risk of secondary infections and complications.^60,61^ Thus, corticosteroids are not recommended for the single treatment of COVID 19.

## CONVALESCENT PLASMA TRANSFUSION

Convalescent plasma transfusion is not a new concept and is being used over a century in the prevention and treatment of many infectious diseases.^62^ Convalescent plasma therapy was found successful in the treatment of Severe Acute Respiratory Syndrome (SARS), Middle East Respiratory Syndrome (MERS), H1N1 pandemic of 2009 and no serious adverse effects were reported with the use of convalescent plasma transfusion.^64-66^ From a meta-analysis of 32 studies on SARS coronavirus infection and severe influenza , it was found that there was significant reduction in mortality after the use of convalescent plasma transfusion compared to placebo and use of no therapy.^67^

A pilot study on convalescent plasma carried out among severe patients of COVID-19 showed potential therapeutic effect along with adequate safety. It was observed in the study that one dose of convalescent plasma with a high concentration of neutralizing antibodies can improve the clinical outcomes of COVID-19 patients by decreasing the viral load promptly.^62^

A systematic review showed improvement in clinical symptoms decreasing the mortality of severe COVID 19 patients with the transfusion of convalescent plasma.^68^

In an observational study carried out among 1315 COVID patients for 3 months period in Nepal revealed highest recovery rate with the combine use of Remdesivir and convalescent plasma in life threatening infections followed by use of Remdesivir alone and least recovery rate was observed with the use of convalescent plasma alone.^32^

Recently, the use of convalescent plasma therapy is not recommended for COVID 19 management in Nepal due to absence of compelling outcomes and findings.^69^

## VACCINES

Following the COVID 19 pandemic, efforts to develop effective vaccines against COVID 19 went all around the world. More than a dozen vaccines were proposed against COVID 19 and many are under the way. Through comprehensive study and review on available preliminary data, World Health Organization has authorized following five vaccines for emergency use so far:^70^

Pfizer-BioNTech vaccine: It is a nucleoside modified mRNA virus manufactured by Pfizer-BioNTech. It was the first vaccine to receive approval for Emergency Use by WHO. It is found to be 95% efficacious against symptomatic SARS-CoV-2 infection. This vaccine was approved for Emergency Use by WHO on 31^st^ December, 2020.

Moderna vaccine: It is mRNA vaccine manufactured by ModernaTX, Inc. and is recommended for use among 18 years and above age group. Its efficacy has been found to be 92% in protecting against COVID 19. It received approval for Emergency use by WHO on 25^th^ January, 2021.

Oxford/AstraZeneca vaccine: Two versions of Oxford/ AstraZeneca vaccine have been listed by WHO for Emergency use: AstraZeneca COVID-19 Vaccine (manufactured by AstraZeneca) and COVISHIELD (manufactured by Serum Institute of India). These are ChAdOx1-S recombinant vaccines developed by AstraZeneca and Oxford University.

Janssen COVID vaccine: It is a viral vector type vaccine manufactured by Janssen Pharmaceuticals Companies of Johnson & Johnson. It was approved for Emergency use by WHO on 17th March 2021. It is recommended for use among 18 years and above age groups.

Vero cell vaccine: It is an inactivated COVID 19 vaccine manufactured by Beijing Bio-Institute of Biological Products Co Ltd, subsidiary of China National Biotec Group (CNBG) under Sinopharm. It has been approved recently on 7^th^ May 2021 for Emergency Use by WHO. The efficacy of this vaccine is found to be 79% for symptomatic and hospitalized disease.

In Nepal, four vaccines have been approved for emergency use. ie. Covishield, Vero cell, Covaxin and Sputnik vaccine.^71-74^ However, only two vaccines "Covishield'' manufactured by Serum Institute of India and "Vero cell" manufactured by Beijing Bio-Institute of Biological Products Co Ltd, subsidiary of China National Biotec Group (CNBG) under Sinopharm have been used so far.

## DISCUSSION

This literature review and analysis was conducted on the basis of recently published studies on the treatment of COVID-19 diseases. This review clearly demonstrates that the available data are not sufficient to recommend any treatment for the elimination of COVID-19 to be used at the clinical level. SARS-CoV-2 was found to emerge from bat and Pangolins, snakes and turtles have been suspected as intermediate hosts of SARS-CoV-2 between bats and human.^75,76^ It is cumbersome to produce SARS-CoV-2 vaccines and antiviral agents because of the novel virus and its nature to undergo a range of mutations. In order to develop effective vaccines and drugs to fight the SARS-CoV-2 pandemic, several studies and investigations are underway worldwide. Altogether, five vaccines have been approved for Emergency use by WHO till now^70^ and other vaccines are also on the way.

Since, development of vaccines and new medicines require huge investment of money, time and effort, to combat the immediate pandemic initially scientists repurposed old medicines.^77^ Several antiviral agents, anti-malarial medicines and analogues were repurposed based on in-vitro data however, clinical studies found no benefit of using anti-malarial drugs, chloroquine in COVID-19.^55,56^ Several clinical trials were carried out to observe the effectiveness of antiviral agents like Ritonavir, Lopinavir, Remdesivir, Favipiravir, Arbidol against SARS-CoV-2. Among these antiviral agents, some in-vitro studies found Remdesivir, Favipiravir and arbidol showing significant effect against SARS-CoV-2.^19-22^ Thus, several clinical trials on Remdesivir and Favipiravir took profound interest on later days of the pandemic.

Studies showed that Remdesivir could block replication of corona viruses. Remdesivir was also found to increase the rate of recovery in hospitalized patients and 5 days course of Remdesivir was beneficial against 10 days course.^26-28^ Another antiviral agent, Favipiravir was found to be effective in day 7 for improving clinical recovery rate among moderate COVID-19 patients than Arbidol. While, Arbidol was found to be effective in improving clinical recovery rate among severe COVID-19 patients in day 7 compared to Favipiravir.^39^ Similarly, an observational study in Nepal showed good outcome among COVID patients with the use of Remdesivir. Owing to all these facts, Remdesivir has been approved for Emergency use in Nepal for COVID treatment; however, only designated hospitals of Nepal have been authorized for its use.^32^ It is being used in hospitalized patient with COVID infection in Nepal based on the disease severity and patient condition. Similarly, clinical trials of Favipiravir among mild to moderate COVID 19 patients are on-going in Nepal. From these findings it can be concluded that Favipiravir could be a potential therapeutic agent for mild to moderate COVID-19 patients and Arbidol could be a potential anti-viral agent for severe COVID-19 patients. Similarly, Remdesivir could be effective in hospitalized patients to reduce the severity. However, despite good outcomes in some studies, arbidol use is seen to be neglected in the COVID 19 management.

Successful use of convalescent plasma in many diseases has a long history.^62-66^ Based on the evidence from decades; convalescent plasma therapy has also been proposed for management of COVID-19 pandemic. Some studies have found significant reduction in mortality with the use of convalescent plasma in COVID-19.^62^ The observational study carried out in Nepal showed improved clinical outcome with the combined use of convalescent plasma and Remdesivir among serious COVID patients. However, there is need for evidence from clinical trials about the therapeutic efficacy of convalescent plasma. The use of convalescent plasma has been recently discontinued in the COVID 19 treatment guideline of Nepal.^69^

Unlike other countries, Nepal is also adopting different measures and using different medicines for the treatment and management of COVID-19 pandemic. Nepal started Convalescent plasma therapy to severe COVID-19 patients in February, 2020 and were carried out in 52 hospitals of Nepal. Similarly, on August, 2020 observational study of Remdesivir among 1315 severe COVID-19 patients requiring supplemental were conducted for 3 months. Recently, trial on Favipiravir among mild to moderate COVID-19 patients is going on in Nepal. In addition to this, other medicines depending upon the cases severity and symptoms are being used in the treatment of COVID 19 in Nepal. Apart from medicines, vaccination campaign are also being conducted in Nepal. "Covishield" vaccines and "Vero Cell" vaccines are being used in Nepal. Studies on adverse effect of vaccines following their administration are being conducted in Nepal.^72,74^

Articles reviewed in this study were obtained from google scholar and pubmed only. Findings of this review were based largely on available literature, documents and guidelines. Much of the documents released were from research performed in China. Clinical studies in medicines and vaccines against COVID 19 are on-going worldwide including Nepal. However, an appropriate treatment regimen to cure COVID 19 could not be established till now.

## CONCLUSIONS

Extensive efforts are being carried out worldwide to develop appropriate medicines and vaccines against COVID 19 from the past one year. Some vaccines and medicines have been approved for Emergency use based on preliminary findings by the country and are being used against COVID 19 infection. Despite these entire global endeavours, COVID 19 still seems to be an indecipherable enigma.
